# Influence of the Weight of a School Backpack on Spinal Curvature in the Sagittal Plane of Seven-Year-Old Children

**DOI:** 10.1155/2015/817913

**Published:** 2015-08-27

**Authors:** Katarzyna Walicka-Cupryś, Renata Skalska-Izdebska, Maciej Rachwał, Aleksandra Truszczyńska

**Affiliations:** ^1^Department of Medicine, Institute of Physiotherapy, University of Rzeszów, Warszawska 26A Street, 35-205 Rzeszów, Poland; ^2^Physiotherapy Department, Józef Piłsudski University of Physical Education, Marymoncka 34, 00-968 Warsaw, Poland

## Abstract

The aim of the paper was to determine a correlation between the weight of a child's backpack, their body weight, and certain features of their body posture. *Material and Methods*. The study group consisted of 109 children, all aged seven years. The parameters of body posture were determined using the Zebris Ultrasonic System. *Results*. The number of children carrying a school backpack in accordance with recommendations was 44 subjects (40.37%). Statistically significant changes were found in the total length of the spine (*Z* = 2.223, *p* = 0.026) and between backpack weight and changes in the following parameters: the total length of the spine (*r*_*s*_ = −0.3999, *p* = 0.017), the length and the angle of the lumbar lordosis (*r*_*s*_ = −0.3352, *p* = 0.049), the angle of the lumbar lordosis (*r*_*s*_ = −0.5065, *p* = 0.002), and the sacral angle (*r*_*s*_ = −0.4279, *p* = 0.010). *Conclusions*. Wearing a backpack heavier than 10% of one's body weight can cause shallowing of the lumbar lordosis and a tendency towards a vertical position of the sacrum. Monitoring the weight of children's school backpacks and enabling them to leave books and notebooks at school would probably be beneficial in reducing the daily burden put on children's spines.

## 1. Introduction

The problem of bad posture in children and adolescents is still a current issue, frequently addressed in scientific publications [1–4]. During the early school period (seven to 10 years), body growth is relatively stable [5]. Kyphotic and balanced body postures dominate during the period of seven to eight years of age [6]. However, when the child begins to attend school, their time spent in a sitting position is extended, which can result in disorders of posturogenesis. Hence, this period is called “the first critical period of posturogenesis.”

Children at this age are exposed to a number of factors that predispose them to the occurrence of bad posture. One of these factors is carrying a backpack that is too heavy for them. According to the literature, this problem affects between 40% and 70% of children in developed countries [5, 7]. Excessive backpack load causes back pain and spinal deformities in children [8–10]. The pain associated with carrying a backpack is referred to as “backpack syndrome.” This syndrome includes the following factors: abnormal body posture causing headaches, fatigue, and cervical and lumbar pain [11, 12].

Recommendations concerning the weight of school backpacks in relation to body weight diverge depending on the organization. In 2009, the American Occupational Therapy Association (AOTA) and the American Physical Therapy Association (APTA) recommended not carrying a backpack heavier than 15% (or between 10% and 20%) of the student's body weight; in 2012, this was changed to 10% of their body weight [13]. The American Chiropractic Association (ACA) recommended that backpack weight should not exceed 5–10% of the child's body weight. There are dangers that excessive loads pose to the maturing spine [14]. Many authors have concluded that the weight of a school backpack should not exceed 10% of the child's body weight [15], based on the fact that it can affect their spinal posture, foot shape, and gait [16–19]. However, there is still no clear information about the impact that a school backpack has on the formation of spinal curvature in the sagittal plane in school children.

The weight of backpacks carried as part of everyday activity may be related to the shape of curvatures of the spine, in particular when the activity requires taking a specific, forced posture. A child carrying a heavy backpack will tend to lean forward to balance their centre of gravity, which results in a reduction of lumbar lordosis and increased thoracic kyphosis [4]. Such a posture may become habitual and be maintained even after taking the backpack off. Thus, the authors of this study decided to look more closely into this issue.

The following hypotheses were set up: does the weight of the backpack constitute the recommended 10% of the body weight of a seven-year-old child? Does curvature of the spine change in children who carry a backpack heavier than 10% of their body weight?

The aim of the paper was to describe the relationship between the load of a child's school backpack and the occurrence of changes in the parameters of their body posture in the sagittal plane.

## 2. Material and Methods

The study was conducted at the end of the first year of primary school at Wincenty Pol Primary School in Lesko, Poland. The study group included 109 children, all aged seven years: 58 girls and 51 boys. The children had not attended a reception class before. The children in the study group were carrying their backpacks on their way to school and back and going from one classroom to another during breaks, for an average total time of 50 minutes a day. The anthropometric data of the children are summarized in Table 1.

The study was conducted after obtaining consent from the school principal, parents, and the subjects themselves and as part of a larger research project which had been approved by the local Bioethics Committee at the Faculty of Medicine, University of Rzeszów.

Inclusion criteria were as follows: only school children who were seven years of age; lack of comorbidities; only traditional school backpacks (i.e., the children's own backpacks which they carried to school every day, designed to be carried on both shoulders).

Exclusion criteria were as follows: children aged younger than seven or over eight years (e.g., children from the reception class and Year 2); lack of consent to participate in research; musculoskeletal, vision, and neurological disorders.

**Figure 1:**
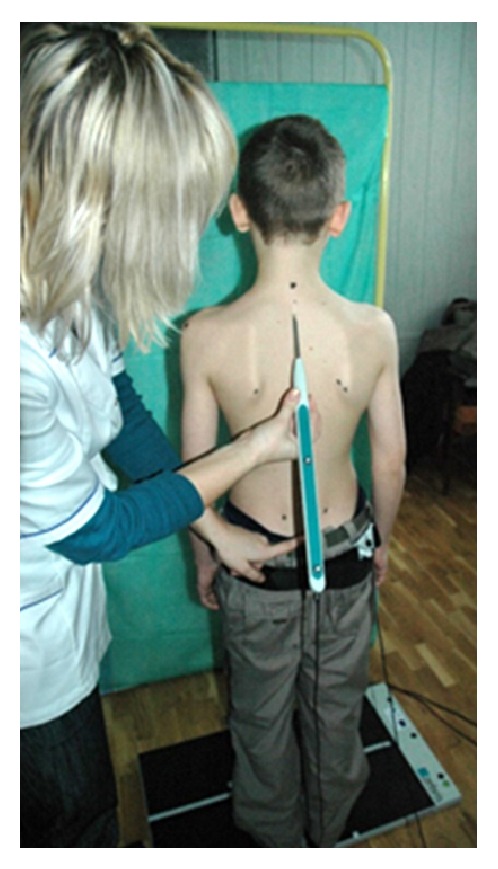
Subject's position during the examination.

The parameters of body posture were determined using the Zebris Ultrasonic System with WinSpine 2.3 software. This system enables three-dimensional analysis of body posture. It consists of a sensor with a stand, the basic CMS-HS unit, and a single marker with a belt to be attached to the hips of the subject. Tests using the ultrasonic spot indicator function by identifying the anatomical landmarks on the subject's skeleton, which are precisely displayed on a monitor connected to the computer [20–22]. Before the start of each test, the following anthropometric points were marked on the skin of the subject: the spinous processes of the spine, the right and left acromion, the right and left anterior superior iliac spine, the right and left iliac crest, the point of change of the thoracic spine into the lumbar spine, and the lower right and left angle of scapula (Figure 1) [23]. The tests were performed by a physiotherapist with five years of experience as an operator of the equipment and 15 years of experience in testing body posture.

The subject was positioned 80 cm from the receiver and equipped with the transmitter belt. The position of the subject was habitual, with the upper limbs along the trunk and the lower limbs extended in the knee and hip joints. The following instructions were given: “stand in a comfortable manner”; “do not bend your knees”; and “look straight.” The children were not instructed to straighten up. When the subject corrected their body posture, the measurement was repeated so that functional defects were also noted [24, 25].

**Figure 2:**
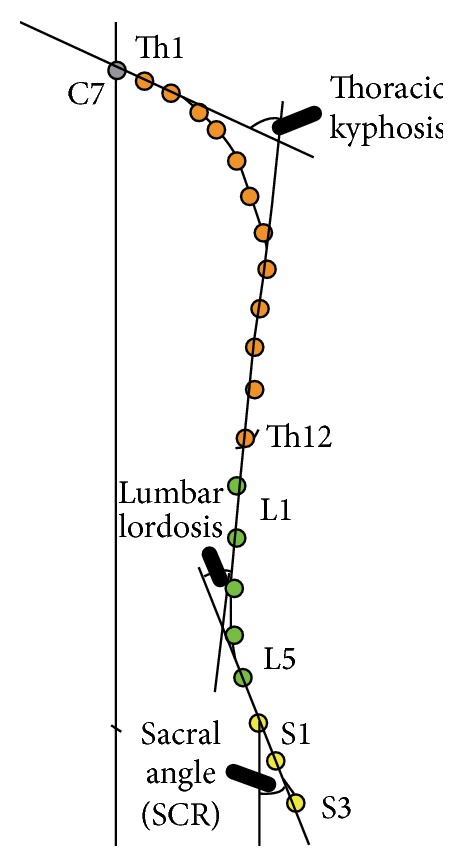
Analysed parameters.

**Figure 3:**
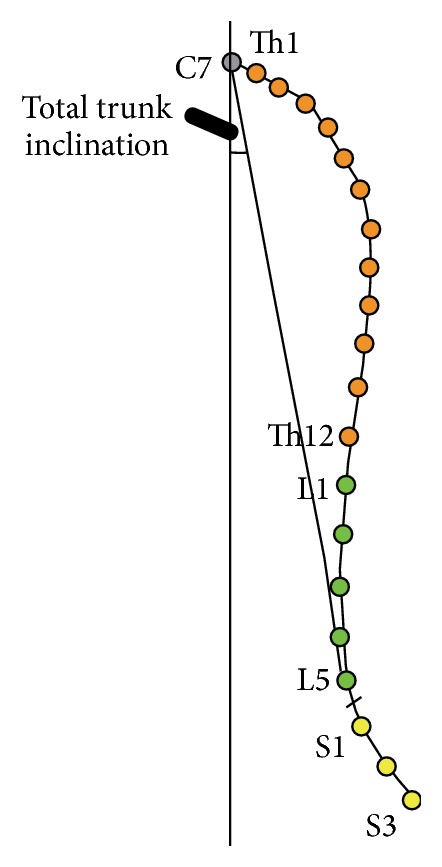
Analysed parameters.

The following parameters of the subjects' posture were measured: ThS (mm): total length of the spine; THL (mm): length of the thoracic spine; LS (mm): length of the lumbar spine; KKP (degrees): thoracic kyphosis angle, calculated from the intersection of the tangents extending between the spinous processes of Th1 and Th2 and Th11 and Th12 (Figure 2); KKL (degrees): angle of lumbar lordosis, calculated from the intersection of the tangents extending between the spinous processes of L1 and L2 and L5 and S1 (Figure 2); TTI (degrees): total angle of anterior trunk inclination (Figure 3); and SCR (degrees): sacral angle in the study group (sacral angle is defined as the angle between the line connecting the spinous processes S1 and S3 and the frontal plane) (Figure 2).

As a next step, the child's body height, body weight, and school backpack weight (along with its contents) were measured. These measurements were made using the seca 213 portable stadiometer height rod (digital scale with 0.1 kg accuracy). The group was examined in the morning, during school hours, that is, during an ordinary school day according to the school timetable.

## 3. Statistical Analysis

The differences in posture between the groups (Group 1, weight of backpack less than 10% of child's body weight, and Group 2, backpack heavier than 10% of child's body weight) were determined using the Mann-Whitney *U* test, which is equivalent to nonparametric variance. The correlation between the weight of the backpack carried by the child every day and the parameters of posture in the sagittal plane was investigated using Spearman's correlation coefficient. The results were considered statistically significant at *p* < 0.05. The obtained results were analysed statistically using STATISTICA 9.0.

## 4. Results

The number of children carrying school backpacks in accordance with recommendations (i.e., weighing less than 10% of their body weight) was 44 subjects (40.37%).

The weight analysis of the school backpacks and their relation to the body weight of the children showed that the weight of the carried load often exceeded the 10% recommended in the literature. Detailed results are shown in Table 2.

The parameters describing the spinal shapes of the two groups (Group 1, with backpacks weighing less than 10% of the children's body weight, and Group 2, with backpacks heavier than 10% of the children's body weight) are shown in Table 3. The only statistically significant differences between the groups were observed for the total length of the spine (*p* = 0.026).

A statistically significant relationship was revealed between the load of the school backpack and the total length of the spine, the length of the lumbar lordosis, the lumbar lordosis angle, and the angle of sacrum inclination. With the increase in the weight of the backpack, the measured parameters decreased. No statistically significant differences were found for the following parameters: THL (length of the thoracic spine), KKP (angle of thoracic kyphosis), and TTI (total angle of anterior trunk inclination). Details are shown in Table 4.

## 5. Discussion

The issue of school backpack load is still a valid one [26, 27]. The new conditions under which children begin to undertake their education may contribute to the emergence or worsening of bad posture. Children's spontaneous physical activity is restricted during the mandatory school age. Their body posture is influenced not only by the restricted room for movement but also by carrying external loads. Overloaded school backpacks can be a threat to the correct development of posture [28].

Children usually carry their backpacks for a relatively short period every day. Nevertheless, the relationship between backpack load and spine shape in the sagittal plane observed in the study arouses a certain interest. In our study, the school backpacks filled with books weighed on average 2.87 kg. The backpacks were weighed once during the study, assuming that the backpack weight would be similar every day, based on the number of daily lessons; these data correspond with the findings of other researchers. Nevertheless, the issue should be taken into account when analysing the obtained results. It has already been mentioned that the results are consistent with the results of other researchers, for example, those of Al-Hazzaa regarding similarly aged children. In this study, the mean weight of the backpack was 2.77 kg, but higher values were accepted for school backpack weight as a percentage of mean body weight (12.5%) compared to in our study (10.9%) [29]. In other parts of the world, higher values have also been recorded, such as 13.2% [30] and 12% [31]. Our study also confirmed this disturbing phenomenon: during the tests carried out in Year 1, the overload of school backpacks was found among 60% of the respondents.

A study by Kułaga and Grajda found that a backpack weight exceeding 10–15% of the child's body weight forces the child to compensate for the excessive load by tilting their torso forward. However, according to the authors, a greater impact on the children's posture was produced by the wrong position of the body, rather than the weight of the backpack [24]. Pau et al. found that an external load increased the distribution of asymmetries in foot pressure forces on the ground. In addition, carrying a school backpack in the wrong way was found to exacerbate existing disorders [32].

Relationships between a child's school backpack load and the total length of their spine, the size of their lumbar lordosis, and their sacrum inclination were all found in our study. When the school backpack weight was higher, greater decrease of the lumbar lordosis was observed, and the sacrum inclination was smaller. Such a situation may lead to a reduction in the natural curvature of the lumbar spine and related adverse consequences, such as reduced amortization properties of the spine, and asymmetric loading of intervertebral discs, which can lead to overloading and degenerative changes in the spine.

There was no relationship observed between backpack load and the length of the thoracic spine, the angle of thoracic kyphosis, or the anterior inclination of the trunk. The lack of correlation with the angle of thoracic kyphosis in our study may be due to the fact that over 40% of the children wore backpacks according to the standard, that is, weighing less than 10% of the weight of the child. The habitual posture during rest and learning should be controlled, and the weight of the school backpacks should also be checked by parents and representatives of the educational institutions. Continuous monitoring and systematic screening tests to detect abnormalities in body posture are also justified.

## 6. The Value of the Research

Studies evaluating the incidence of abnormal behaviour, such as children wearing heavy backpacks and the relationship between this and their posture, are important in the prevention of back pain. The observed reduction and shortening of the lumbar spine as the weight of the carried backpack increases may indicate a primary cause of subsequent future overloading and degenerative changes in the spine. It can also help explain the occurrence of lower back pain in school children, discussed widely in the literature [33–35]. The unambiguous evidence showing that the weight of school backpacks is related to the shape of spine curvature will allow schools to include compensation exercises in physical education curricula, ensuring optimum physical development by encouraging balanced and spontaneous physical activity during the day (e.g., as recreation activities). Thus, opportunities to help protect the spine will be created.

## 7. Limitations of the Research

The weight of the backpacks without content (which would have indicated the actual weight of the school books and notebooks) was not examined, as the weight was checked only once. In addition, the study was not a longitudinal study conducted over a longer period, for example, at the beginning and the end of the school year.

## 8. Conclusions

- Wearing a backpack heavier than 10% of one's body weight can cause shallowing of the lumbar lordosis and a tendency towards a vertical position of the sacrum.
- Monitoring the weight of children's school backpacks by parents and teachers, as well as children themselves, and enabling them to leave books and notebooks at school would probably be beneficial in reducing the daily burden put on children's spines.

## Figures and Tables

**Table 1:** Basic biometric parameters characterizing the study group (*N* = 109).

	Mean	Range	SD
Body weight (kg)	25.55	19.02–38.00	4.7
Body height (m)	1.25	1.13–1.39	0.1

SD: standard deviation.

**Table 2:** The weight of the school backpacks carried by the subjects.

	Mean	Range	SD
Weight of school backpack (kg)	2.87	1.64–4.10	0.50

Weight of school backpack in relation to child's body weight (%)	10.90	6.78–17.47	3.14

SD: standard deviation.

**Table 3:** Summary of the parameters characterizing the children's body postures.

	Backpacks lighter than 10% of body weight			Backpacks heavier than 10% of body weight			Mann-Whitney *U* test	
	Mean	Range	SD	Mean	Range	SD	*Z*	*p*
ThS (mm)	375.4	350.3–400.5	25.1	357.0	320.7–393.3	36.3	2.223	0.026
THL (mm)	230.4	212.5–248.3	17.9	223.4	203.7–243.1	19.7	1.252	0.211
LS (mm)	107.0	90.6–123.4	16.4	98.4	77.7–119.1	20.7	1.454	0.146
KKP (deg.)	47.4	33.8–61.0	13.6	43.8	31.1–56.5	12.7	1.414	0.157
KKL (deg.)	26.6	12.3–40.9	14.3	23.9	12.2–35.6	11.7	1.112	0.266
TTI (deg.)	4.2	2.3–6.1	1.9	3.4	1.4–5.4	2.0	1.345	0.179
SCR (deg.)	24.5	14.7–34.3	9.8	19.5	9.3–29.7	10.2	1.927	0.054

ThS (mm): total length of the spine.

THL (mm): length of the thoracic spine.

LS (mm): length of the lumbar spine.

KKP (degrees): thoracic kyphosis angle, calculated from the intersection of the tangents extending between the spinous processes of Th1 and Th2 and Th11 and Th12.

KKL (degrees): angle of lumbar lordosis, calculated from the intersection of the tangents extending between the spinous processes of L1 and L2 and L5 and S1.

TTI (degrees): total angle of anterior trunk inclination.

SCR (degrees): sacral angle in the study group (sacral angle is defined as the angle between the line connecting the spinous processes S1 and S3 and the frontal plane).

SD: standard deviation; *Z*: Mann-Whitney *U* test; *p*: significance level.

**Table 4:** The relationship between the child's school backpack load and the occurrence of changes in the parameters of the child's body posture in the sagittal plane, using Spearman's correlation coefficient.

Anthropometric parameters	*r*_*s*_, *p* (value)	Interpretation
ThS	−0.3999, (0.017)	The heavier the backpack, the lower the total length of the spine

THL	−0.2313, (0.181)	Not significant

LS	−0.3352, (0.049)	The heavier the backpack, the lower the length of the lumbar lordosis

KKP	−0.2695, (0.117)	Not significant

KKL	−0.5065, (0.002)	The heavier the backpack, the smaller the lumbar lordosis angle

TTI	−0.0537, (0.759)	Not significant

SCR	−0.4279, (0.010)	The heavier the backpack, the smaller the sacral angle

ThS (mm): total length of the spine.

THL (mm): length of the thoracic spine.

LS (mm): length of the lumbar spine.

KKP (degrees): thoracic kyphosis angle.

KKL (degrees): lumbar lordosis angle.

TTI (degrees): total angle of anterior trunk inclination.

SCR (degrees): sacral angle.

*r*_*s*_: Spearman's correlation coefficient.
